# Chronic stress enhances microglia activation and exacerbates death of nigral dopaminergic neurons under conditions of inflammation

**DOI:** 10.1186/1742-2094-11-34

**Published:** 2014-02-24

**Authors:** Rocío M de Pablos, Antonio J Herrera, Ana M Espinosa-Oliva, Manuel Sarmiento, Mario F Muñoz, Alberto Machado, José L Venero

**Affiliations:** 1 Department of Biochemistry and Molecular Biology, Faculty of Pharmacy, University of Seville, E-41012 Seville, Spain; 2 Institute of Biomedicine of Seville, Virgen del Rocio University Hospital/CSIC/University of Seville, E-41013 Seville, Spain; 3 Present address: Gray Institute for Radiation Oncology and Biology, Department of Oncology, University of Oxford, Oxford OX3 7LJ, UK

**Keywords:** Glucocorticoids, Lipopolysaccharide, Microglia, Parkinson’s disease, Stress, Substantia nigra

## Abstract

**Background:**

Parkinson’s disease is an irreversible neurodegenerative disease linked to progressive movement disorders and is accompanied by an inflammatory reaction that is believed to contribute to its pathogenesis. Since sensitivity to inflammation is not the same in all brain structures, the aim of this work was to test whether physiological conditions as stress could enhance susceptibility to inflammation in the substantia nigra, where death of dopaminergic neurons takes place in Parkinson’s disease.

**Methods:**

To achieve our aim, we induced an inflammatory process in nonstressed and stressed rats (subject to a chronic variate stress) by a single intranigral injection of lipopolysaccharide, a potent proinflammogen. The effect of this treatment was evaluated on inflammatory markers as well as on neuronal and glial populations.

**Results:**

Data showed a synergistic effect between inflammation and stress, thus resulting in higher microglial activation and expression of proinflammatory markers. More important, the higher inflammatory response seen in stressed animals was associated with a higher rate of death of dopaminergic neurons in the substantia nigra, the most characteristic feature seen in Parkinson’s disease. This effect was dependent on glucocorticoids.

**Conclusions:**

Our data demonstrate that stress sensitises midbrain microglia to further inflammatory stimulus. This suggests that stress may be an important risk factor in the degenerative processes and symptoms of Parkinson’s disease.

## Background

Parkinson’s disease (PD) is an age-related neurodegenerative disorder characterised by progressive degeneration of the nigrostriatal dopaminergic (DAergic) neurons of the substantia nigra pars compacta (SNpc) [1]. This process results in extrapyramidal motor dysfunction accompanied by progressive impairment of autonomy, mood and cognitive function [1-3]. Although some genes have been identified as being responsible for rare familial early-onset PD [4], the aetiology of PD remains elusive. Oxidative stress, reduced expression of trophic factors, mitochondrial dysfunction, alterations of the ubiquitin proteasome system and neuroinflammatory mechanisms are thought to collaborate in the progressive demise of SNpc neurons [1,2,5-11].

Evidence suggesting that inflammation may play a central role in the cell loss in PD has been accumulating since the presence of activated microglia in the substantia nigra (SN) of PD patients was first reported ( [12]; for review, see [13,14]). The increased number of activated microglial cells is accompanied by increased expression of proinflammatory cytokines [6,15]. Inflammation has been shown to exist in different animal models of PD, including those using 1-methyl-4-phenyl-1,2,3,6-tetrahydropyridine (MPTP), 6-hydroxydopamine (6-OHDA) or rotenone [16-19]. Lipopolysaccharide (LPS) is the active immunostimulant in the cell wall of Gram-negative bacteria that is responsible for triggering the cascade of events following bacterial infection [20,21]. Subtoxic doses of LPS exacerbated disease progression in an animal model of PD [22], supporting the hypothesis that brain inflammation may play a significant role in PD progression. More important, epidemiological studies have demonstrated that the incidence of idiopathic PD is about 50% lower in chronic users of nonsteroidal anti-inflammatory drugs and cyclooxygenase inhibitors than in age-matched nonusers [23-25].

Our group pioneered in showing that an inflammatory response induced by the intracerebral injection of LPS can cause neuronal death, specifically in the nigrostriatal DAergic system [26-28]. Interestingly, the SN, compared with other brain areas, is especially susceptible to LPS-induced neurotoxicity [28]. In this structure, the strong microglial response to LPS preceded the death of DAergic neurons [26,29]. This point has been confirmed by experiments with chronic infusion of LPS into the SN that produced delayed death of DAergic neurons, as also found in PD [17]. Under these conditions, microglial activation reached a plateau 2 weeks earlier than the appearance of degenerative events on the DAergic system [17]. The neurotoxic effect of LPS has been also observed in mesencephalic cell cultures [30-33].

Some physiological conditions might enhance the inflammatory response to LPS and account for the diversity in symptoms and course of PD, as well as for individuals’ responses to medication after the onset of PD [34]. Stress is widely acknowledged to be a predisposing and precipitating factor in psychiatric illnesses [35,36] and some neurodegenerative diseases. It was one of the earliest proposed causes of PD [37,38]. Stress is a condition of human experience that includes not only major life events but also the hassles of daily life that elevate activities of physiological systems, which causes disruption of ongoing homeostasis. It reflects both individual experience and genetic background; therefore, reactions to a stressful incident are highly variable [39,40]. Exposure to a stressful situation leads to the activation of two systems: the sympathoadrenomedullary system and the hypothalamic-pituitary-adrenal (HPA) axis (for review, see [41]). The former leads to increased circulating levels of adrenaline, whereas the latter leads to release of corticosteroid hormones from the adrenal cortex. Corticosteroid hormones can easily enter the brain because of their lipophilic properties and bind two types of receptors: mineralocorticoid receptors (MRs) and glucocorticoid receptors (GRs) (for review, see [42]). Whereas MRs have a high affinity for the endogenous hormone corticosterone, GRs have a lower affinity. Interestingly, MRs are restricted mainly to limbic regions, including the amygdala and all subareas of the hippocampus. On the contrary, GRs are ubiquitously distributed in neurons and glia. Although stress can be beneficial in its acute phase, repeated and severe stressful stimuli produce adverse effects on neuronal functions, especially in those structures involved in the stress response, such as the hypothalamus, prefrontal cortex (PFC) and hippocampus.

We have previously shown a synergistic deleterious effect of chronic stress and inflammation in limbic structures such as the hippocampus [43] and the PFC [44]. These studies suggest that stress strongly sensitises microglial cells to proinflammatory stimuli. However, whether this effect is generalised in the whole central nervous system (CNS) is unknown. The aim of the present work was to elucidate whether enhancement of the LPS-induced damage by chronic stress is extensible to other CNS structures, especially to those involved in neurodegenerative disorders such as PD, in which inflammation seems to play an important role. To evaluate this hypothesis, we tested whether chronic variate stress could enhance the damage induced by LPS in the SN. We combined immunohistochemical and molecular biological techniques in an effort to elucidate the effects of chronic stress, intranigral LPS injection and a combination of both on different cell types in the SN. We found that stress significantly increased the inflammatory damage induced by LPS. We also studied the effect produced on the different parameters assayed by RU486 (mifepristone (11β-[*p*-(dimethylamino)phenyl]-17β-hydroxy-17-(1-propynyl)estra-4,9-dien-3-one)), a potent inhibitor of GR activation.

## Methods

### Animals and surgery

**Figure 1:**
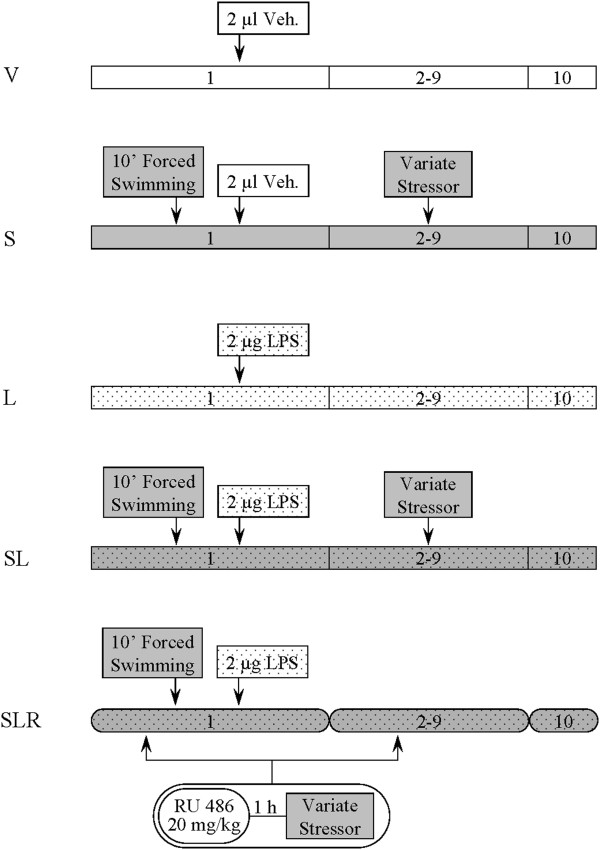
**Experimental groups and treatments.** Intranigral injections of vehicle (Veh) or lipopolysaccharide (LPS) were given at day 1. In the stressed groups (S, SL and SLR), intranigral injections were carried out after the application of the first stressor (10 minutes of forced swimming). In the SLR group, RU486 (mifepristone (11β-[*p*-(dimethylamino)phenyl]-17β-hydroxy-17-(1-propynyl)estra-4,9-dien-3-one)) was injected subcutaneously every day 1 hour before the stressor. V, vehicle; S, stress; L, lipopolysaccharide; SL, lipopolysaccharide injected into stressed animals; SLR, lipopolysaccharide injected into stressed animals treated with RU486.

Male albino Wistar rats (250 to 270 g) were used for these studies. The rats were kept at constant room temperature of 22°C ± 1°C and 60%relative humidity on a 12:12-hour light–dark cycle with free access to food and water. Rats were anaesthetized with chloral hydrate (400 mg/kg) and positioned in a stereotaxic apparatus (Kopf Instruments, Tujunga, CA, USA) to conform to the brain atlas of Paxinos and Watson [45]. Injections into the SN were made 5.5 mm posterior, 1.8 mm lateral and 8.3 mm ventral to the bregma at day 1, 2 hours after the application of the first stressor (Figure 1).

Experiments were carried out in accordance with the Guidelines of the European Union Council (86/609/EU) and Spanish regulations for the use of laboratory animals (BOE 67/8509-12, 1988), and the study was approved by the Scientific Committee of the University of Seville.

Five groups of animals were established according to the different treatments: V, the vehicle/nonstressed control group, which received a single intranigral injection of 2 μl of vehicle (Monastral Blue inert tracer, 1% in saline solution; Sigma-Aldrich, St Louis, MO, USA) into the left SN; S, the vehicle/stressed group, which were treated with a single intranigral injection of 2 μl of vehicle into the left SN and were stressed for 9 days; L, the LPS group (nonstressed animals), which were treated with a single intranigral injection of 2 μg of LPS (from *Escherichia coli* serotype 026:B6; Sigma-Aldrich) dissolved in 2 μl of vehicle (1% Monastral Blue inert tracer in saline solution) into the left SN; SL, the LPS/stressed group, which were treated with a single intranigral injection of 2 μg of LPS and stressed for 9 days; SLR, the LPS/stressed/RU486 group, which were treated with a single intranigral injection of 2 μg of LPS, stressed for 9 days and were given a daily dose of 20 mg/kg RU486 in saline with 20% dimethyl sulphoxide by subcutaneous injection (Sigma-Aldrich) for 9 days 1 hour before exposure to the stressors. All animals were killed by decapitation at 6 hours after surgery (RT-PCR experiments) or by perfusion 10 days after surgery (immunohistochemistry experiments). At least four animals were used for each group.

### Stress model

Chronic variate stress was adapted from other models of variate stress [46-51] with modifications as reported previously [43,44]. Animals were divided into stressed and nonstressed groups. Nonstressed animals were kept undisturbed in their home cages for 10 days. A 9-day variate stressor paradigm was used for the animals in the stressed groups. The schedule of stressors is given in Table 1. Application of stress started at different times from day to day (between 08:00 and 20:00) to minimize its predictability. Restraint was carried out by placing each animal in a 21 cm × 6 cm plastic tube and adjusting it with plaster tape on the outside, so that the animal was unable to move. There was a 6-cm hole at the far end for breathing. Forced swimming was carried out by placing the animal in a glass tank measuring 44 × 33 × 30 cm with 22 cm of water depth at 23°C ± 2°C. Body weight was measured at the beginning and the end of the 10-day treatment and was evaluated as an indirect parameter of HPA axis activation.

**Table 1:** **Schedule of stressors used during the chronic variate stress treatment**^**a**^

**Day**	**Stressor**	**Time**
1	Forced swimming	10 min
2	Restraint	3 h
3	Water deprivation	24 h
4	Restrain at 4°C	90 min
5	Isolation	24 h
6	Food deprivation	24 h
7	Water deprivation	24 h
8	Restrain at 4°C	2 h
9	Food deprivation	24 h

^a^Animals subjected to stress were exposed to the stressors according to the schedule listed. The left column indicates the day of the stressing protocol. The right column indicates the length of each stimulus applied.

### Serum corticosterone measurement

Rats were deeply anaesthetized, and blood was collected from the heart. Serum corticosterone concentration was measured by enzyme-linked immunosorbent assay according to the manufacturer’s instructions (Assay Designs Correlate-EIA; Enzo Life Sciences, Farmingdale, NY, USA).

### Immunohistological evaluation

Thaw-mounted 20-μm coronal sections were cut on a cryostat at -15°C and mounted on gelatine-coated slides. Primary antibodies used were rabbit-derived anti-tyrosine hydroxylase (1:300 anti-TH; Sigma-Aldrich), mouse-derived anti-glial fibrillary acidic protein (1:300 anti-GFAP; EMD Millipore, Billerica, MA, USA), rabbit-derived anti-Iba-1 (1:300; Wako Chemicals, Richmond, VA, USA) and mouse-derived OX-6 (1:200; AbD Serotec, Raleigh, NC, USA). Incubations and washes for all the antibodies were carried out in Tris-buffered saline (TBS), pH 7.4. All work was done at room temperature. Sections were washed and then treated with 0.3% hydrogen peroxide in methanol for 20 minutes, washed again, and incubated in a TBS solution containing 1% horse serum (Vector Laboratories, Burlingame, CA, USA) for GFAP and OX-6 immunostaining or in a solution containing goat serum (Vector Laboratories) for TH and Iba-1 immunostaining for 60 minutes in a humidity chamber. Slides were drained and further incubated with the primary antibody in TBS containing 1% horse or goat serum and 0.25% Triton X-100 for 24 hours. Sections were then incubated for 2 hours with biotinylated horse anti-mouse immunoglobulin G (IgG, 1:200; Vector Laboratories) for GFAP and OX-6 immunostaining or biotinylated goat anti-rabbit IgG (1:200; Vector Laboratories) for TH and Iba-1 immunostaining. The secondary antibody was diluted in TBS containing 0.25% Triton X-100, and its addition was preceded by three 10-minute rinses in TBS. Sections were then incubated with ExtrAvidin–Peroxidase buffered aqueous solution (1:100; Sigma-Aldrich). The peroxidase was visualized by performing a standard diaminobenzidine–hydrogen peroxide reaction for 5 minutes.

### Immunohistochemical data analysis

Analyses were carried out in a bounded region of the SN with a length of 300 μm in the anteroposterior axis centred at the point of injection (5.5 mm with respect to the bregma), that is, between 5.35 and 5.65 mm with respect to the bregma. In each case, five sections per animal were used with random starting points and systematically distributed through the anteroposterior axis of the analysed region. For the measurement of areas lacking GFAP immunoreactivity, we used analySIS imaging software (Soft Imaging System GmbH, Münster, Germany) coupled to a Polaroid DMC camera (Polaroid, Cambridge, MA, USA) attached to a Leica light microscope (Leica Microsystems, Wetzlar, Germany). To count cells showing OX-6 immunoreactivity, we systematically sampled the area occupied by the OX-6-positive cells in each section from a random starting point with a grid adjusted to count five fields per section. An unbiased counting frame of a known area (40 × 25 μm = 1,000 μm^2^) was superimposed on the tissue section image under a 100× oil immersion lens objective. The different types of OX-6-positive cells (displaying different shapes, depending on their activation state) were counted as a whole and expressed as cells per millimetre squared. The number of TH-positive neurons in the SN was estimated using a fractionator sampling design [52]. Counts were carried out at regular predetermined intervals within each section (*x* = 150 μm and *y* = 200 μm). An unbiased counting frame of known area (40 μm × 25 μm = 1,000 μm^2^) was superimposed on the tissue section image under a 100× oil immersion lens objective. Therefore, the area sampling fraction was 1,000/(150 × 200) = 0.033. The entire *z*-dimension of each section was sampled; hence, the section thickness sampling fraction was 1. In all animals, 20-μm sections, each 100 μm apart, were analysed; thus, the fraction of sections sampled was 20/100 = 0.20. The number of neurons in the analysed region was estimated by multiplying the number of neurons counted within the sample regions by the reciprocals of the area sampling fraction and the fraction of section sampled.

### Immunofluorescence

Animals were perfused and sections prepared as described above. Incubations and washes for all the antibodies were carried out in phosphate-buffered saline (PBS), pH 7.4. All work was done at room temperature. For double-labelling of Iba-1 with TH, sections were blocked with PBS containing 1% normal horse serum (Vector Laboratories) for Iba-1 and 1% goat serum (Vector Laboratories) for TH for 1 hour. The slides were washed three times in PBS, then incubated overnight at 4°C with either mouse-derived anti-Iba-1 (1:300; EMD Millipore) and rabbit-derived anti-TH (1:300; Sigma-Aldrich) diluted in PBS containing 1% normal horse serum and 1% goat serum and 0.25% Triton X-100. Sections were incubated with horse anti-mouse secondary antibody conjugated to fluorescein (1:200; Vector Laboratories) for Iba-1 and goat anti-rabbit secondary antibody conjugated to Alexa Fluor 594 (1:200; Molecular Probes/Invitrogen, Eugene, OR, USA) for TH for 1 hour at 22°C ± 1°C in the dark, and their addition was preceded by three 10-minute rinses in PBS. Nuclei were counterstained with Hoechst dye (1 μg/ml; Molecular Probes). As a control, another set of experiments was performed whereby the sections were incubated with only the Iba-1 antibody, then visualized with both fluorescence filters. No signal was detected when Iba-1 alone was used with an Alexa Fluor 594 filter (photomicrograph not shown). The same was true with TH when a fluorescein filter was used.

For double-labelling of Iba-1 with inhibitor of nuclear factor κB (NF-κB) kinase, subunit β (IKKβ), sections were blocked with PBS containing 1% normal goat serum (Vector Laboratories) for Iba-1 and rabbit serum (Invitrogen) for TH for 1 hour. The slides were washed three times in PBS, then incubated overnight at 4°C with either rabbit-derived anti-Iba-1 (1:300; Wako Chemicals) and goat-derived anti-IKKβ (1:300; Santa Cruz Biotechnology, Santa Cruz, CA, USA) diluted in PBS containing 1% normal goat and 1% rabbit serum and 0.25% Triton X-100. Sections were incubated with goat anti-rabbit secondary antibody conjugated to Alexa Fluor 594 (1:200; Molecular Probes/Invitrogen) for Iba-1 and with rabbit anti-goat secondary antibody conjugated to fluorescein (Vector Laboratories; 1:200) for IKKβ for 1 hour at 22°C ± 1°C in the dark. Their addition was preceded by three 10-minute rinses in PBS. Nuclei were counterstained with Hoechst dye (1 μg/ml; Molecular Probes/Invitrogen). As a control, another set of experiments was performed whereby the sections were incubated with only the Iba-1 antibody, then visualized with both filters. No signal was detected when Iba-1 alone with the fluorescein filter was used (photomicrograph not shown). The same was true for IKKβ when an Alexa Fluor 594 filter was used. Fluorescence images were acquired using a Zeiss LSM 7 DUO confocal laser scanning microscope (Carl Zeiss Microscopy, Jena Germany) and processed using the associated software package (ZEN 2010; Carl Zeiss Microscopy).

### Real time RT-PCR

The left SN was dissected from each rat 6 hours after the injection of vehicle or LPS, snap-frozen in liquid nitrogen and stored at -80 °C. Total RNA was extracted from the SN using the RNeasy kit (QIAGEN, Germantown, MD, USA). cDNA was synthesized from 1 μg of total RNA using the QuantiTect Reverse Transcription Kit (QIAGEN) in 20 μl of reaction volume as described by the manufacturer. Real-time PCR was performed with iQ SYBR Green Supermix (Bio-Rad Laboratories, Hercules, CA, USA), 0.4 μM primers and 1 μl of cDNA. Controls were carried out without cDNA. Amplification was run in a MasterCycler ep *realplex* thermal cycler (Eppendorf, Happauge, NY, USA) at 94°C for 3 minutes, followed by 35 cycles at 94°C for 30 seconds, 55°C to 60°C for 45 s and 72°C for 45 s, then by a final elongation step at 72°C for 7 minutes. Following amplification, melting curve analysis was performed by heating the reactions from 65°C to 95°C at 1°C intervals while monitoring fluorescence. Analysis confirmed a single PCR product at the predicted melting temperature. β-actin served as the reference gene and was used for sample normalization. Primer sequences for tumour necrosis factor α (TNF-α), interleukin 6 (IL-6), IL-1β, inducible nitric oxide synthase (iNOS), CD200, chemokine (C-X3-C motif) receptor 1 (CX3CR1), monocyte chemoattractant protein 1 (MCP-1) and β-actin are shown in Table 2. The cycle at which each sample crossed a fluorescence threshold cycle (C_t_) was determined, and the triplicate values for each cDNA were averaged. Analyses of real-time PCR were carried out using a comparative C_t_ method integrated into Bio-Rad system software.

**Table 2:** **Primers used for RT-PCR**^**a**^

**Target mRNA**	**Forward (F) and reverse (R) primers**	**Reference**
TNF-α	F: 5′-TACTGAACTTCGGGGTGATTGGTCC-3′	[53]
	R: 5′-CAGCCTTGTCCCTTGAAGAGAACC-3′	
IL-6	F: 5′-AAAATCTGCTCTGGTCTTCTGG-3′	[54]
	R: 5′-GGTTTGCCGAGTAGACCTCA-3′	
β-actin	F: 5′-TGTGATGGTGGGAATGGGTCAG-3′	[55]
	R: 5′-TTTGATGTCACGCACGATTTCC-3′	
IL-1β	F: 5′-CAGGATGAGGACATGAGCACC-3′	[55]
	R: 5′-CTCTGCAGACTCAAACTCCAC-3′	
iNOS	F: 5′-CCTCCTCCACCCTACCAAGT-3′	[56]
	R: 5′-CACCCAAAGTGCTTCAGTCA-3′	
CD200	F: 5′-TGTTCCGCTGATTGTTGGC-3′	[57]
	R: 5′- ATGGACACATTACGGTTGCC-3′	
CX3CR1	F: 5′-GGC CTT GTC TGA TCT GCT GTT TG-3′	[58]
	R: 5′- AAT GCT GAT GAC GGT GAT GAA GAA-3′	
MCP-1	F: 5′-AGCATCCACGTGCTGTCTC-3′	Universal ProbeLibrary^b^
	R: 5′-GATCATCTTGCCAGTGAATGAG-3′	

^a^*CX3CR1*, chemokine (C-X3-C motif) receptor 1; *IL-6*, Interleukin 6; *iNOS*, inducible nitric oxide synthase; *MCP-1*, monocyte chemoattractant protein 1; *TNF*, Tumour necrosis factor. ^b^Roche Applied Science (Indianapolis, IN, USA).

### Western blot analysis

SN was lysed in 15 mM Tris–HCl, pH 7.5, 150 mM NaCl, 1 mM ethylenediaminetetraacetic acid, 1 mM ethylene glycol tetraacetic acid and 1 mM phenylmethylsulfonyl fluoride (all from Sigma-Aldrich). The homogenate was centrifuged at 12,000 × *g* for 20 minutes at 4°C. Protein content of the samples was estimated by the method of micro-Lowry using bovine serum albumin as a standard [59], and 25 μg of protein was loaded for each lane. Protein samples were separated by SDS-PAGE (10%) and transferred onto a nitrocellulose membrane (Novex; Life Technologies, Grand Island, NY, USA). Membranes were blocked with blocking buffer (5% milk in TBS: 20 mM Tris–HCl, pH 7.5, 500 mM NaCl and 0.05% Tween 20) for 1 hour at room temperature. Membranes were then incubated using anti-cyclooxygenase 2 (anti-Cox-2) antibody (1:1,000; Santa Cruz Biotechnology). β-actin antibody (Sigma-Aldrich; 1:2,000) was used as a loading control.

### Determination of lipid hydroperoxide by oxidation of Fe^2+^ in presence of xylenol orange

The protocol for lipid peroxidation measurements [60] was adapted for a microplate reader. Sample buffer (10 μl) was incubated with 90 μl of H_2_SO_4_ for 30 minutes. After addition of 100 μl of FOX reagent (0.5 mM ferrous ammonium sulphate, 200 mM sorbitol and 0.2 mM xylenol orange in 25 mM H_2_SO_4_), the mixture was incubated at room temperature for 45 minutes while protected from light. The formation of ferric ions was detected by measuring the resulting coloured complex formed by xylenol orange at 560 nm (ε = 4.3 × 10^4^/M/cm).

### Statistical analysis

Results are expressed as mean ± SD. Means were compared by using Student’s *t*-test (weight gain and serum corticosterone levels) or analysis of variance (ANOVA) followed by the least significant difference (LSD) test for *post hoc* multiple range comparisons. The α value was set at 0.05. STATGRAPHICS Plus 3.0 software was used for the calculations (Statpoint Technologies, Warrenton, VA, USA).

## Results

### Validation of the stress model

**Figure 2:**
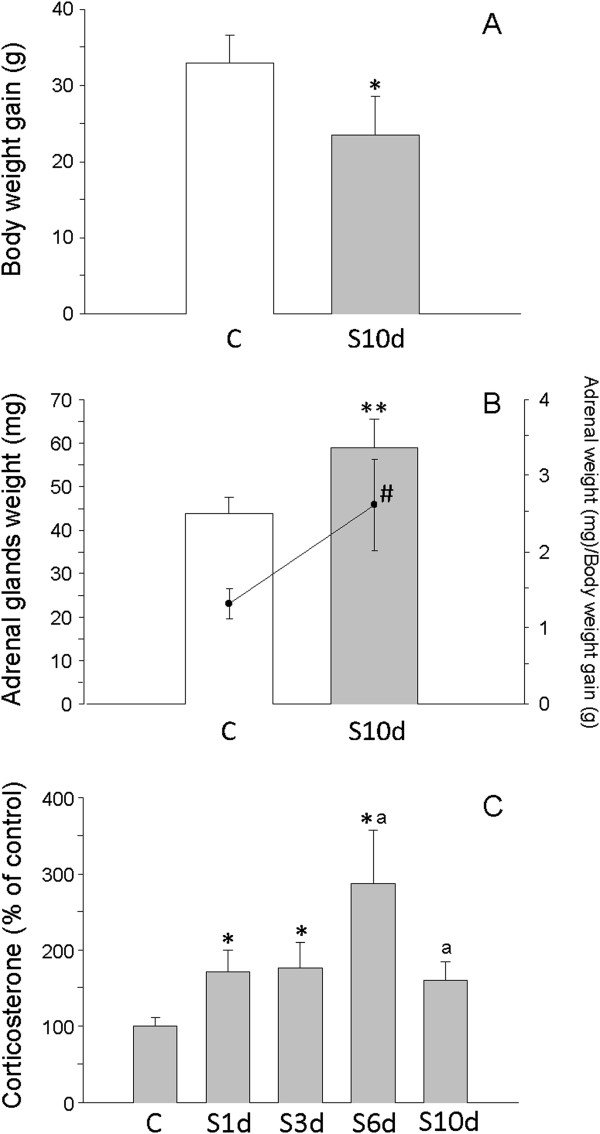
**Validation of the stress model. (A)** Body weight gain (in grams). *P < 0.05. **(B)** Adrenal gland weight (in milligrams; bars) and ratio between adrenal gland weight and body weight gain (scatterplot and line). **P < 0.01, ^#^*P* < 0.01 (for adrenal gland weight/body weight gain ratio). **(C)** Serum corticosterone (percentage of control animals). *P < 0.01 compared with control, ^a^*P* < 0.01 compared with previous time point (S1d to S10d indicates days subjected to variate stress). Statistical significance was calculated by using Student’s *t*-test to compare data before **(C)** and after 10 days (S10d) of variate stress. Data were derived from one-way analysis of variance followed by the least significant difference (LSD) *post hoc* test for multiple range comparisons.

Body weight gain alteration is a typical effect of stress and is used as a method to assess stress models [46]. In our experimental design, stressed animals gained 28.8% less weight than controls (*P* < 0.05) (Figure 2A). Adrenal gland weight increased 34.7% in stressed animals (*P* < 0.01) (Figure 2B). The ratio of adrenal gland weight to body weight gain increased by 94.0% in the stressed animals compared with the controls (*P* < 0.01) (Figure 2B). Serum levels of corticosterone were evaluated before stress and at days 1, 3, 6 and 10 of the variate stressor paradigm. Because steroids levels are subjected to daily changes, blood samples were collected between 09:00 and 10:00. As expected, there was a significant increase in corticosterone levels that peaked at day 6 in stressed animals and decreased afterward (Figure 2C).

### Activated microglia quantification and expression levels of TNF-α, IL-6, IL-1β and iNOS mRNAs and COX-2 protein

**Figure 3:**
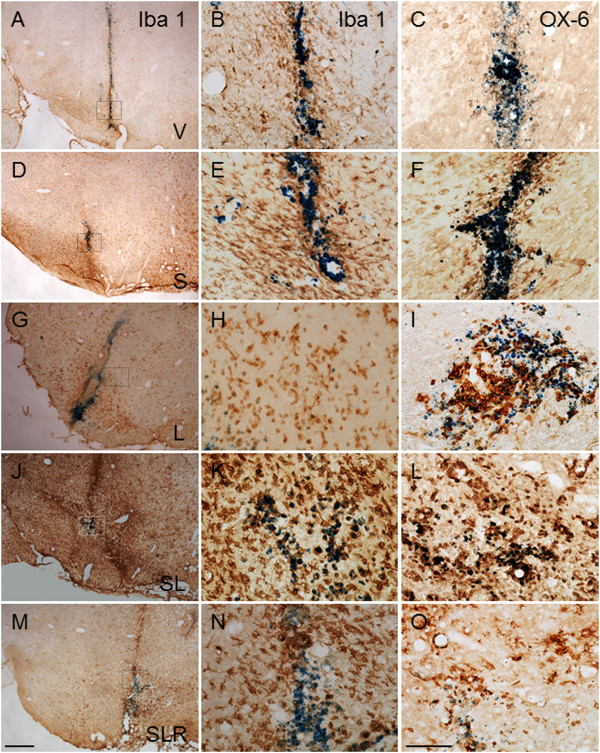
**Effect of chronic stress on the lipopolysaccharide-induced activation of microglia in the ventral mesencephalon.** Midbrain microglia were evaluated by immunohistochemistry with Iba-1 (left and middle columns) and OX-6 antibodies (right column) in vehicle-injected animals **(A)** through **(F)** and lipopolysaccharide (LPS)-injected animals **(G)** through **(L)** under nonstressed conditions (**(A)** through **C)** and **(G)** through **(I)**) and stressed conditions (**(D)** through **(F)** and **(J)** through **(L)**). Iba-1 immunohistochemistry is shown at low and high magnification (left and middle columns, respectively). **(M)** through **(O)** The effect of RU486 (mifepristone (11β-[*p*-(dimethylamino)phenyl]-17β-hydroxy-17-(1-propynyl)estra-4,9-dien-3-one)) on the microglia population in response to LPS injection in stressed animals. Note that stress highly increased the microglial activation response to LPS injection **(J)** through **(L)** compared with nonstressed conditions **(G)** through **(I)**. Note that, after LPS injection, most microglial cells display a round morphology typical of macrophages, whose density significantly increases under conditions of chronic stress. Also note how RU486 treatment strongly prevents the stress-induced sensitisation of microglia to subsequent LPS injection **(M)** through **(O)**. The blue staining in all panels is the Monastral Blue inert tracer contained in the vehicle. Scale bars: 500 μm **(A**, **D**, **G**, **J and M)**; 100 μm (all other panels). Abbreviations: V, Vehicle; S, Stress; SL, Lipopolysaccharide injected into stressed animals; SLR, Lipopolysaccharide injected into stressed animals treated with RU486.

**Figure 4:**
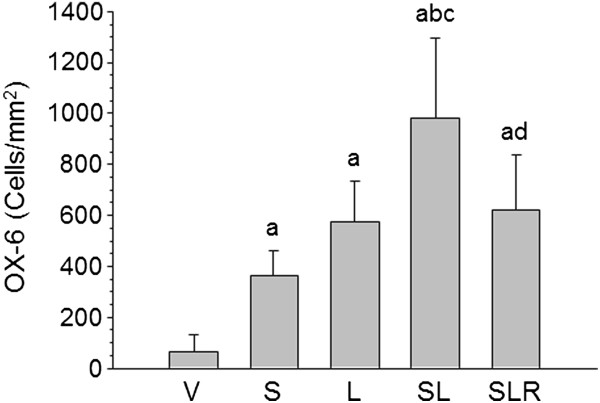
**Effect of lipopolysaccharide and stress on the number of activated microglial cells in the substantia nigra.** Quantification of changes on the reactive microglial population in the substantia nigra at the end of the treatments. Results are mean ± SD of four independent experiments expressed as OX-6-positive cells/mm^2^. *P* < 0.001 by one-way analysis of variance followed by the least significant difference *post hoc* test for multiple range comparisons: a, compared with vehicle (V); b, compared with stress (S); c, compared with lipopolysaccharide (L); d, compared with stress + lipopolysaccharide (SL). SLR, Lipopolysaccharide injected into stressed animals treated with RU486 (mifepristone (11β-[*p*-(dimethylamino)phenyl]-17β-hydroxy-17-(1-propynyl)estra-4,9-dien-3-one)).

Microglial cells become activated (that is, change of morphology from resting resident ramified microglia with two or three fine processes to round cells resembling tissue macrophages) and proliferate when challenged. Immunohistochemistry of Iba-1 and OX-6 showed that injection of vehicle in nonstressed animals (controls) produced a slight microglial reaction that was more intense in stressed animals (approximately fivefold that of controls; *P* < 0.001) (Figures 3 and 4). LPS injection induced stronger activation in nonstressed animals (about eightfold compared with control animals; *P* < 0.001). Combination of stress and LPS produced an additive effect (nearly 15-fold increase over controls; *P* < 0.001) that was reduced by RU486 (decreased ninefold from control values; *P* < 0.001).

**Figure 5:**
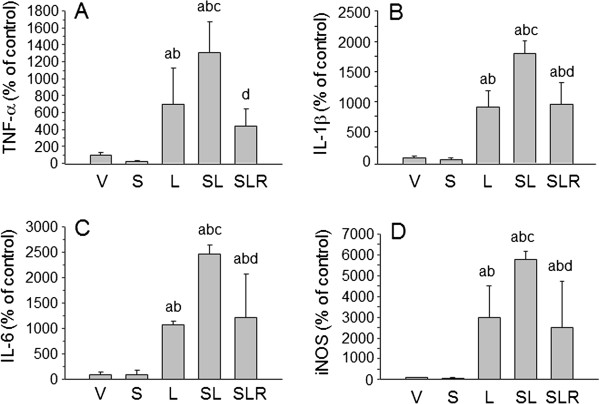
**Effect of lipopolysaccharide and stress on expression of tumour necrosis factor α, interleukin 1β, interleukin 6 and inducible nitric oxide synthase mRNAs in substantia nigra.** mRNA expression was quantified by real-time RT-PCR. Stress had no effect in the vehicle-injected animals. As expected, lipopolysaccharide (LPS) injection increased the expression levels of mRNA. This induction was higher when LPS and stress were combined, whereas treatment with RU486 (mifepristone (11β-[*p*-(dimethylamino)phenyl]-17β-hydroxy-17-(1-propynyl)estra-4,9-dien-3-one)) prevented this effect. Results are mean ± SD of at least three independent experiments expressed as percentage of control values. Statistical significance was calculated by one-way analysis of variance followed by the least significant difference *post hoc* test for multiple range comparisons a, compared with vehicle (V); b, compared with stress (S); c, compared with lipopolysaccharide (L); d, compared with stress + lipopolysaccharide (SL). SLR, Lipopolysaccharide injected into stressed animals treated with RU486 (mifepristone (11β-[*p*-(dimethylamino)phenyl]-17β-hydroxy-17-(1-propynyl)estra-4,9-dien-3-one)). **(A)** Tumour necrosis factor α (TNF-α), *P* < 0.001. **(B)** Interleukin 1β (IL-1β), *P* < 0.001. **(C)** Interleukin 6 (IL-6), *P* < 0.01. **(D)** Inducible nitric oxide synthase (iNOS), *P* < 0.01.

Microglia in the SN were also studied at the molecular level by RT-PCR to evaluate their activation under stress, brain inflammation or both. Activated microglia produce various cytokines and proinflammatory substances with different actions, depending on the inductor of their activation. Previous short-term temporal analysis revealed that the highest induction of cytokines occurred 6 hours after the LPS injection and decreased at 48 hours [61]. Hence, we measured the differences in the expression profile of the cytokines in the ventral mesencephalon at 6 hours after the injection of LPS. Stress produced no effect on TNF-α (Figure 5A), whereas LPS induced an increase in its expression in nonstressed animals sevenfold over control values (*P* < 0.001). When LPS was injected into stressed animals, expression levels of TNF-α were about 13-fold greater than control values (*P* < 0.001). The expression levels of IL-1β in SN were not affected by stress (Figure 5B) and increased after the injection of LPS in both nonstressed and stressed animals (approximately 9- and 18-fold greater than control values, respectively; *P* < 0.001). Similarly, the expression levels of IL-6 in SN were not affected by stress (Figure 5C) and increased after the injection of LPS in both nonstressed and stressed animals (about 10- and 25-fold above control values, respectively; *P* < 0.05). The expression levels of iNOS in SN were not affected by stress (Figure 5D) and increased after the injection of LPS in both nonstressed and stressed animals (approximately 30- and 60-fold over control values, respectively; *P* < 0.05). RU486 reduced the expression levels of all parameters assayed to values close to those induced by LPS in nonstressed animals.

The protein levels of COX-2 were measured by Western blot analysis. As expected, LPS treatment increased the expression levels of COX-2, by 33.1% (*P* < 0.05). Stress induced a nonsignificant tendency to further increase the protein levels of COX-2 in both vehicle- and LPS-injected animals (data not shown).

**Figure 6:**
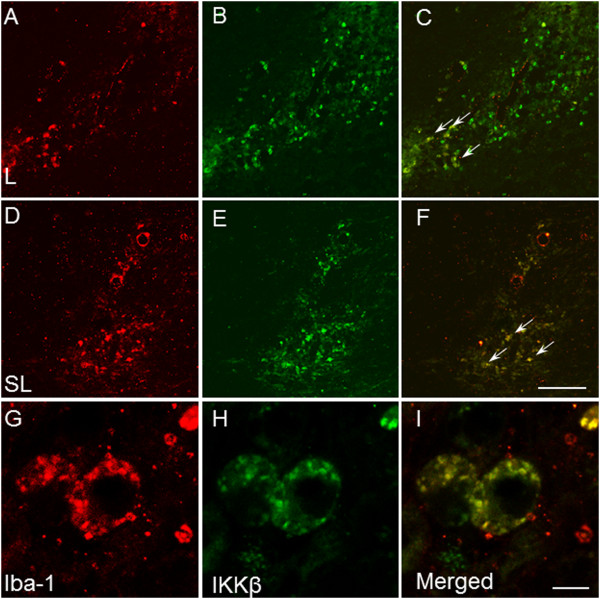
**Effect of stress and lipopolysaccharide on inhibitor of nuclear factor κB kinase, subunit β, and Iba-1 in the substantia nigra.** Iba-1 immunofluorescence **(A)**, **(D)** and **(G)** and inhibitor of nuclear factor κB kinase, subunit β (IKKβ), immunofluorescence **(B)**, **(E)** and **(H)** show robust induction of IKKβ in Iba-1-labelled microglial cells in the merged images **(C)**, **(F)** and **(I)**. Images **(A)**, **(B)** and **(C)** were taken of an animal injected with lipopolysaccharide (LPS; L). Images **(D)** through **(F)** were taken of a stressed animal injected with LPS (SL). Images **(G)**, **(H)** and **(I)** are high-magnification photomicrographs showing one representative cell. Scale bars: **(A)** through **(F)**, 200 μm; **(G)** through **(I)**, 25 μm. Arrows indicate colocalizing cells.

In order to obtain direct proof that microglia are activated in a proinflammatory state and release proinflammatory mediators, we performed double-immunofluorescence with Iba-1 and IKKβ. As shown in Figure 6, most activated microglial cells colocalized with IKKβ in the ventral mesencephalon.

### Effect lipopolysaccharide and stress on lipid peroxidation

**Figure 7:**
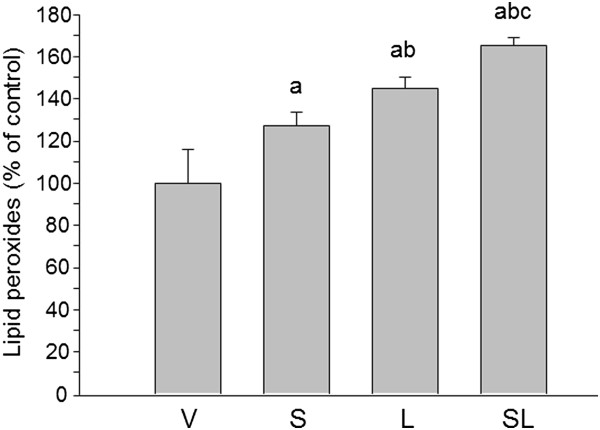
**Effect of stress and lipopolysaccharide on lipid peroxidation in the substantia nigra.** Lipid peroxidation increased in stressed animals as well as after the injection of lipopolysaccharide (LPS) in nonstressed animals. When combined, stress and LPS had an additive effect. *P* < 0.01 by one-way analysis of variance followed by least significant difference *post hoc* test for multiple range comparisons. a, compared with vehicle (V); b, compared with stress (S); c, compared with lipopolysaccharide (L). SL, stressed animals injected with lipopolysaccharide.

To investigate the production of reactive oxygen species (ROS) induced by stress, we determined lipid peroxidation using a FOX assay. As shown in Figure 7, stress increased the amount of lipid peroxides in both vehicle- and LPS-injected animals (127 ± 6.18% and 165 ± 3.48% of control values; *P* < 0.001).

### Quantification of CD200, CX3CR1 and MCP-1 mRNA expression levels

**Figure 8:**
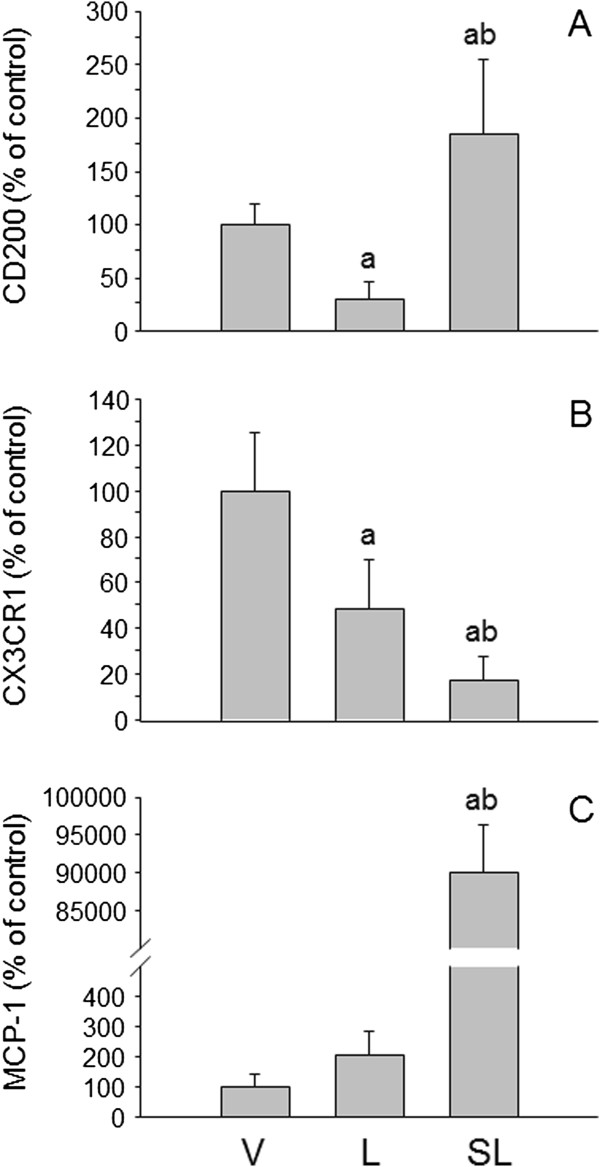
**Effect of stress and lipopolysaccharide on the expression of CD200, chemokine (C-X3-C motif) receptor 1 and monocyte chemoattractant protein 1 mRNAs in the substantia nigra.** mRNA expression was quantified by real-time RT-PCR. **(A)** CD200. Lipopolysaccharide (LPS) decreased CD200 expression in nonstressed animals and increased it in stressed rats. **(B)** Chemokine (C-X3-C motif) receptor 1 (CX3CR1). LPS decreased CX3CR1expression in nonstressed animals, and in stressed animals the decrease was even greater. **(C)** Monocyte chemoattractant protein 1 (MCP-1). No significant change was observed after injection of LPS into nonstressed rats. However, the effect of LPS on MCP-1 expression in stressed animals was massive. *P* < 0.01 by one-way analysis of variance followed by least significant difference *post hoc* test for multiple range comparisons. a, compared with vehicle (V); b, compared with lipopolysaccharide (L). SL, stressed animals injected with lipopolysaccharide.

There is evidence that several molecules associate with inhibitory actions on microglia, including CD200 and CX3CR1. Hence, we decided to monitor these molecules in our experimental conditions to seek further explanations of how stress triggers an exacerbated response. The expression levels of CD200 mRNA in SN were reduced after the injection of LPS into nonstressed animals (29.6 ± 17% of control values; *P* < 0.05) (Figure 8A), whereas these levels were increased after the injection of LPS into stressed animals (185 ± 69.2% of control values; *P* < 0.05). However, the expression levels of CX3CR1 mRNA in SN were reduced after the injection of LPS in both nonstressed and stressed animals (48.7 ± 21.1% and 17.4 ± 10.2% of control values, respectively; *P* < 0.001) (Figure 8B).

The MCP-1–CCR2 chemokine axis is an important mediator of the migration of monocytes, memory T lymphocytes and natural killer cells into affected areas in diseases such as multiple sclerosis, rheumatoid arthritis, type 2 diabetes and Alzheimer’s disease [62,63]. Hence, we decided to study the changes in the expression levels of the chemokine MCP-1 in the SN of stressed and nonstressed animals. Our results show that chronic stress induces a dramatic increase in the mRNA expression of MCP-1 in the SN of LPS-injected animals (*P* < 0.001) (Figure 8C).

### Astroglia population

**Figure 9:**
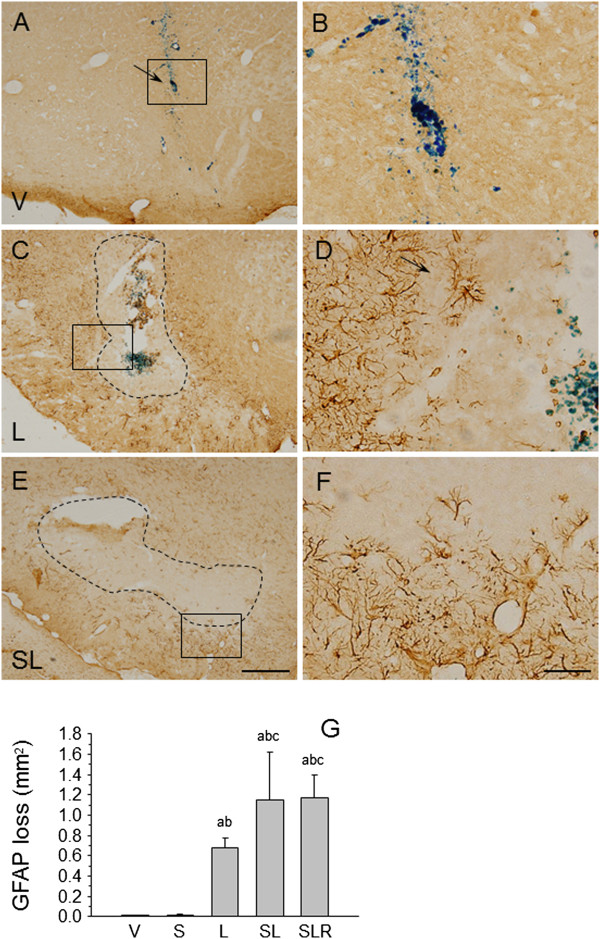
**Effect of stress and lipopolysaccharide in astroglia in the substantia nigra. (A)** Coronal section showing glial fibrillary acidic protein (GFAP) immunoreactivity in a vehicle-injected nonstressed animal (arrow points to injection site). A limited alteration restricted to the needle tract is observed. **(B)** High-magnification image of the area within the box in **(A)**. **(C)** GFAP immunoreactivity in a lipopolysaccharide (LPS)-injected nonstressed animal. There is an area lacking GFAP immunoreactivity around the injection track (dotted encircled area). **(D)** High-magnification image of the square box in **(C)**; the arrow shows the injection site. **(E)** GFAP immunoreactivity in a LPS-injected stressed animal. The area lacking GFAP immunoreactivity is bigger (dotted encircled area). **(F)** High magnification of the square box in panel E showing hypertrophic astrocytes surrounding the injection site. Scale bars: **(A)**, **(C)** and **(E)**, 500 μm; **(B)**, **(D)** and **(F)**, 100 μm. Abbreviations: V, vehicle; S, stress; L, lipopolysaccharide; SL, lipopolysaccharide injected into stressed animals; SLR, lipopolysaccharide injected into stressed animals treated with RU486 (mifepristone (11β-[*p*-(dimethylamino)phenyl]-17β-hydroxy-17-(1-propynyl)estra-4,9-dien-3-one)). **(G)** Quantification of the areas lacking GFAP immunoreactivity on the substantia nigra at the end of the treatments. Results are mean ± SD of at least four independent experiments expressed in millimetres squared. *P* < 0.001 by analysis of variance followed by least significant difference *post hoc* test for multiple comparisons. a, compared with vehicle (V); b, compared with stress (S); c, compared with lipopolysaccharide (L). SL, stressed animals injected with lipopolysaccharide; SLR, lipopolysaccharide injected into stressed animals treated with RU486.

We have previously shown that the intranigral injection of LPS induces the loss of astroglia through a mechanism that is not yet well-known [26-28]. In our present study, we found that there is slight astrogliosis around the vehicle injection site, without loss of GFAP immunostaining, in nonstressed and stressed animals (Figures 9A, 9B and 9G). Astroglia disappeared around the LPS injection site, an area absent of GFAP-positive structures (0.67 mm^2^; *P* < 0.001 compared with control animals) but surrounded by hyperreactive astrocytes (Figures 9C, 9D and 9G). Stress and LPS interact in a synergistic manner, producing an increase in the area lacking astrocytes (1.15 mm^2^; *P* < 0.001 compared with control animals) (Figures 9E, 9F and 9G). This effect was not reduced by RU486 (1.17 mm^2^ (Figure 9G).

### Dopaminergic neurons

**Figure 10:**
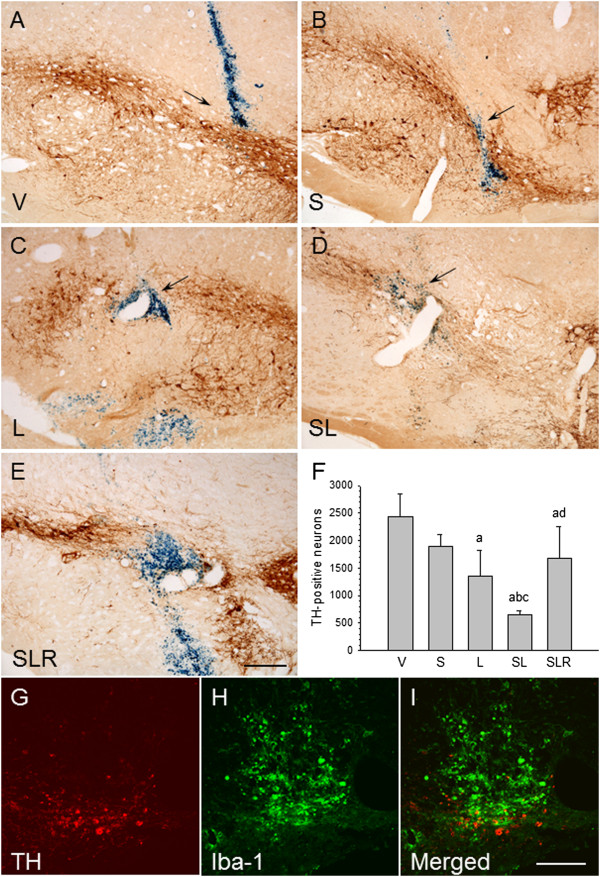
**Chronic stress increases the lipopolysaccharide-induced loss of dopaminergic neurons in the substantia nigra. (A)** Coronal section showing tyrosine hydroxylase (TH) immunoreactivity after the injection of vehicle (arrow) in nonstressed animals. **(B)** TH immunoreactivity after the injection of vehicle (arrow) in stressed animals. No significant changes can be observed. **(C)** TH immunoreactivity after the injection of 2 μg of lipopolysaccharide (LPS) into the substantia nigra (SN) of nonstressed rats. There is a loss of dopaminergic neurons around the injection track (arrow). **(D)** TH immunoreactivity after the injection of 2 μg of LPS into the SN of stressed rats. The loss of neurons is higher around the injection track (arrow). **(E)** RU486 (mifepristone (11β-[*p*-(dimethylamino)phenyl]-17β-hydroxy-17-(1-propynyl)estra-4,9-dien-3-one)) diminished the loss of TH-positive neurons caused by the combined action of LPS and stress. Scale bar: 500 μm. Abbreviations: V, vehicle; S, stress; L, lipopolysaccharide; SL, lipopolysaccharide injected into stressed animals; SLR, lipopolysaccharide injected into stressed animals treated with RU486. **(F)** Quantification of the number of TH-positive cells. Results are mean ± SD of four independent experiments expressed as TH-positive cells within the bounded area of the SN. *P* < 0.001 by analysis of variance followed by least significant difference *post hoc* test for multiple comparisons. a, compared with V; b, compared with S; c, compared with L; d, compared with SL. **(G)** Immunofluorescence of TH after the injection of 2 μg of LPS into the SN of stressed rats. **(H)** Immunofluorescence of Iba-1 after the injection of 2 μg of LPS into the SN of stressed rats. **(I)** Merged image of **(G)** and **(H)** showing activated microglia around the dopaminergic neurons. Scale bars in **(G)** through **(I)**: 100 μm.

TH immunostaining was carried out to detect DAergic neurons. An even distribution was seen in the SN of nonstressed and stressed animals injected with vehicle (Figures 10A and 10B). As we have previously shown, the intranigral injection of LPS produced a decrease in the number of DAergic neurons around the injection site (55.6% of controls; *P* < 0.001) (Figure 10C). When LPS was injected into stressed rats, its effect was stronger, decreasing the number of neurons to 27.1% of that of controls (*P* < 0.001) (Figure 10D). This effect was reduced by RU486 (69.0% of controls; *P* < 0.001) (Figure 10E). In order to demonstrate the presence of morphologically active microglial cells in the areas of less DAergic neuronal density, we performed double-immunostaining of TH and Iba-1 (Figures 10G, 10H and 10I). Our results show that, in the areas with activated microglia, the TH-immunopositive cells are scarce and have a degenerative morphology.

## Discussion

In our present study, we show that chronic stress exacerbates microglial activation after injection of a proinflammatory stimulus such as LPS in the ventral mesencephalon, leading to an increase in the death of DAergic neurons in the SN. This effect was glucocorticoid (GC)-dependent because treatment with the GR antagonist RU486 prevented stress-induced microglial overactivation and the subsequent higher neuronal death in response to LPS.

In a previous study, our group showed that chronic stress strengthened the inflammatory stimulus associated with a single LPS injection in limbic areas such as the PFC, and, more important, induced extensive neuronal loss [44]. The injection of LPS into the PFC induced a moderate inflammatory response compared with injection into the SN [26,44]. Similar results were found when LPS was injected into the hippocampi of stressed animals [43]. However, contrary to the effect observed in the PFC, the hippocampus was totally resistant to the proinflammatory reaction induced by LPS in the absence of stress. Consistently, our study results suggest that stress strongly sensitises microglial cells to proinflammatory stimuli in limbic areas, which express high levels of GR [64]. It is unknown if this is a general effect in the CNS. Consequently, the aim of the present study was to elucidate whether microglial sensitisation by stress in the hippocampus and the PFC is extensible to other CNS structures, especially those involved in neurodegenerative disorders in which inflammation seems to play an important role [65]. To test this hypothesis, we performed intracerebral injection of LPS into the SN, which is characterized by a high density of microglia [66]. This feature makes the SN highly reactive to proinflammatory stimuli. The degeneration of the nigral DAergic system is the most important distinguishing characteristic of PD.

Our study shows that there is an important neurodegenerative process in the SN of stressed animals after the injection of LPS. Stereological analysis revealed a significant effect of chronic stress, reinforcing the loss of TH-immunopositive neurons induced by LPS. In a previous study, Smith *et al*. [67], by using the 6-OHDA model of PD, showed that chronic psychological stress accelerates neural degeneration, suggesting that stress could be an aggravating factor in DAergic degeneration. The question that remains is how chronic stress sensitises DAergic neurons to further damage. To answer this question, we used a model of DAergic degeneration based on brain inflammation in the ventral mesencephalon induced by a single intranigral injection of LPS. Our long-term analysis shows that, when combined, stress and LPS significantly increased microglial activation compared with LPS alone, an indication that stress sensitises microglia in brain areas other than limbic structures such as the hippocampus and the PFC. A typical feature of Toll-like receptor ligation is activation of the transcription factor NF-κB, leading to transcription of proinflammatory genes [65]. Activation of NF-κB relies on activation of the IKK complex, in which IKKβ triggers the canonical pathway. We have previously shown that LPS induces IKKβ expression in reactive microglia similarly to iNOS, as revealed by Western blot analysis and quantitative PCR [68]. In our present study, we performed dual-immunofluorescence detection of Iba-1 and IKKβ in the ventral mesencephalon in response to intranigral LPS injection to further test whether microglia were indeed activated and thus capable of releasing proinflammatory mediators responsible for the death of DAergic neurons. Our immunohistochemical data demonstrate robust induction of IKKβ in Iba-1-labelled microglia in the ventral mesencephalon in response to LPS injection in the absence or presence of chronic stress, thus demonstrating proinflammatory gene expression in LPS-induced reactive microglia. ROS generation is another hallmark of neurotoxic microglia, which readily attack numerous biomolecules, including lipids, nucleic acids and proteins. We wanted to know whether LPS would increase the oxidation status of mesencephalic tissue and whether chronic stress would alter the expected LPS-induced free radical generation. Toward this end, we quantified lipid peroxidation in mesencephalic tissue using a FOX assay. As expected, LPS significantly increased lipid peroxidation, and, more important, chronic stress further increased this effect, in keeping with its deleterious effect on the DAergic system. Real-time PCR shows an increase in the mRNA expression levels of different inflammatory mediators, including IL-6, TNF-α, IL-1β and iNOS, 6 hours after the treatment with LPS, which was further elevated in the LPS-injected animals under chronic stress. These findings are suggestive of microglia priming. When priming does occur in microglia or peripheral macrophages, these sensitised cells do not produce proinflammatory or anti-inflammatory products, but, if further stimulated, they produce high levels of proinflammatory products [69-75].

GCs are generally regarded as anti-inflammatory and indeed have a variety of actions that inhibit inflammation. However, it is known that the temporal relationship between GC treatment and immune challenge may be an important factor in determining whether GCs exhibit pro- or anti-inflammatory properties. To shed light on this issue, we studied the mRNA expression of CD200 and CX3CR1 (also known as fractalkine receptors), which have been associated with inhibitory actions on brain microglia [76], and MCP-1, a potent chemokine which has been implicated in different neurological disorders [77]. CD200 is an extrinsic factor widely expressed not only on neurons but also on astrocytes and oligodendrocytes [78]. Its receptor, CD200R, is expressed exclusively on macrophages in the CNS, including microglia. The interaction of neuronal CD200 with CD200R leads to inactivation of microglia and keeps them in a resting state [79,80]. As expected, intranigral LPS injection led to significant downregulation of CD200 mRNA expression in the ventral mesencephalon. However, chronic stress turned the LPS-induced downregulation of CD200 expression into significant upregulation of this extrinsic microglia regulator, thus excluding CD200 as a physiological stress-related molecule responsible for activating microglia. In the CNS, microglia are the only cells that express CX3CR1 [81]. Cardona *et al*. [81], using CX3CR1^+/-^ and CX3CR1^-/-^ mice, demonstrated an inverse relationship between CX3CR1 expression and neurotoxic activation of microglia in three different models of neurodegeneration, including the MPTP model of PD, a transgenic model of amyotrophic lateral sclerosis, and systemic LPS injections. Our data demonstrate that intranigral LPS injection significantly decreased CX3CR1 expression. Strikingly, chronic stress further strengthened LPS-induced CX3CR1 downregulation. It will be very important to discover whether physiological stress downregulates CX3CR1 expression in different animal models of neurodegeneration, taking into consideration the role of CX3CR1 as a selective regulator of microglial neurotoxicity *in vivo*.

MCP-1 is the most potent activator of signal transduction pathways leading to monocyte transmigration [82]. There is strong evidence that MCP-1 is involved in the recruitment of monocytes, macrophages and activated lymphocytes into the CNS [83]. Of note, chronic stress dramatically increased the LPS-induced expression of MCP-1 in the mesencephalon, which may facilitate the infiltration of peripheral immune cells. In support of this observation, chronic stress has been shown to stimulate the expression of MCP-1 along with infiltration of bone marrow-derived microglia into the paraventricular nucleus [84]. Taken together, our results demonstrate that chronic stress modulates key molecules regulating brain inflammation, which opens the potential for strategies aimed at minimising its deleterious effects.

Frank *et al*. [69] showed that, when corticosterone was administered 2 hours before LPS injection, corticosterone was unable to inhibit hippocampal cytokine responses potentiating the production of IL-6. In our experimental conditions, LPS was injected 2 hours after the introduction of the first stressor (forced swimming). It is known that, immediately after acute forced swimming stress, corticosterone levels increase in plasma [85]. This suggests that stress sensitises (that is, primes) midbrain microglia to subsequent proinflammatory stimuli. Consistent with our results, other studies have shown that exposure to stressors increases the immune activation state in the CNS. For example, Nair and Bonneau [86] showed that chronic restraint stress increased microglial proliferation and microglial activation. Taking into consideration our findings in previous studies on limbic areas [43,44], our present results are consistent with the view that stressors sensitise the neuroinflammatory response to central immunological challenges.

Ros-Bernal *et al*. [87] studied the involvement of GC-GR in MPTP-lesioned mice in terms of DAergic loss and microglial activation. Using conditional GC-CR-knockout mice in the myeloid cell lineage, including microglia and peripheral macrophages, they found higher loss of TH-positive neurons in the SN of knockout mice, as well as higher reactive microgliosis than that in wild-type mice. Further, proinflammatory genes were significantly upregulated in the conditional GC-GR-knockout mice [87]. These findings are good examples of how acute and chronic stress may have opposite effects in terms of brain inflammation and associated neurodegeneration. Our model of chronic stress was validated in three different ways; time course of corticosterone levels, body weight loss and hypertrophy of adrenal glands. Besides, we had previously measured dopamine release in the PFC under the same stress paradigm [44]. Overall, our data demonstrate a persistent elevation of GC (9 days) covering the period from the intranigral LPS injection to the immunohistochemical data analysis. In fact, the most plausible mechanism associated with our chronic stress paradigm is the long-lasting increase in systemic GC associated with activation of the HPA axis. To prove this hypothesis, we tested the ability of RU486 (mifepristone, a potent inhibitor of GR activation and also an antagonist of the progesterone receptor) to counteract the aggravating effect of chronic stress on the LPS-induced death of nigral DAergic neurons. We have used a dose of 20 mg/kg RU486, as we and others have reported previously [43,44,88-90]. Treatment with RU486 significantly protects nigral TH-positive neurons from the damage observed in stressed animals injected with LPS. Importantly, treatment with RU486 prevented the "priming effect" associated with stress in terms of activated microglia and expression of proinflammatory markers in response to intranigral LPS. Altogether, these data show that GCs play a critical role in the activation of microglia in response to LPS, leading to increased neuronal death in the SN.

There is no clear evidence of a causal relationship between stress (and stress hormones) and PD. However, an increasing base of data suggests that they could be an important factor in its pathogenesis. GR density is not equal in all brain structures, and, interestingly, it is higher in regions that are involved in motor control, such as the motor cortex, basal ganglia and cerebellum [91,92]. This makes these areas more susceptible to the effects of stress, so that stress and stress hormones affect the function of intact (that is, undamaged) motor systems in both humans [93] and rats [94,95]. This is also observed in PD patients, with a positive association between cortisol and gait deficits [96]. It has been shown that immobilization stress increases oxidative stress and that this selectively damages the DAergic system [97]. This is consistent with studies showing that the DAergic system is particularly sensitive to stress [98-100]. However, it seems that the greatest risk factor for the development of PD is age, which could contribute to a deterioration of the compensatory mechanisms that extends along with the preclinical stages of the disease [101,102]. Also, there is an alteration in the response to stress during aging. For example, the response of the HPA axis to stress is higher, and its return to homeostatic conditions is slower, so that cells are exposed to high levels of GCs for a longer period (see [103] for a review). The deregulation of these processes could be related to the higher sensitivity of the DAergic neurons to damage, causing neurons of certain brain areas to be more susceptible to degeneration during stress episodes.

## Conclusions

Our data show that chronic stress exacerbates microglial activation and the death of DAergic neurons after an inflammatory challenge to the ventral midbrain, suggesting that stress may be an important factor in the degenerative processes in and the development of symptoms of PD. Therefore, new strategies to reduce the effect of stress should be explored for the prevention and treatment of PD and other neurodegenerative conditions.

## Abbreviations

6-OHDA: 6-hydroxydopamine; CNS: Central nervous system; GC: Glucocorticoid; GFAP: Glial fibrillary acidic protein; GR: Glucocorticoid receptor; HPA: Hypothalamic–pituitary–adrenal axis; IL: Interleukin; LPS: Lipopolysaccharide; MPTP: 1-methyl-4-phenyl-1,2,3,6-tetrahydropyridine; MR: Mineralocorticoid receptor; PD: Parkinson’s disease; PFC: Prefrontal cortex; SNpc: Substantia nigra pars compacta; TH: Tyrosine hydroxylase; TNF: Tumour necrosis factor; TBS: Tris-buffered saline.

## Competing interests

The authors declare that they have no competing interests.

## Authors’ contributions

RMP performed the surgery, carried out the immunohistochemistry and PCR experiments, analysed and interpreted data, and contributed to the writing of the manuscript. AJH contributed to the design of the experiments, analysis of data and writing of the manuscript. AMEO performed the enzyme-linked immunosorbent assays and analysed data. MS carried out the perfusions. MFM carried out Western blots. AM was involved in drafting the manuscript and revising it critically. JLV conceived the study, contributed to its experimental design and coordination and helped with the writing. All authors read and approved the final manuscript.
